# Orchestrating the Dermal/Epidermal Tissue Ratio during Wound Healing by Controlling the Moisture Content

**DOI:** 10.3390/biomedicines10061286

**Published:** 2022-05-31

**Authors:** Alexandru-Cristian Tuca, Ives Bernardelli de Mattos, Martin Funk, Raimund Winter, Alen Palackic, Florian Groeber-Becker, Daniel Kruse, Fabian Kukla, Thomas Lemarchand, Lars-Peter Kamolz

**Affiliations:** 1 Department of Surgery, Division of Plastic, Aesthetic and Reconstructive Surgery, Medical University of Graz, 8036 Graz, Austria; r.winter@medunigraz.at (R.W.); alen.palackic@gmx.at (A.P.); lars.kamolz@medunigraz.at (L.-P.K.); 2 Department Tissue Engineering & Regenerative Medicine (TERM), University Hospital Würzburg, 97080 Würzburg, Germany; ives.de_mattos@uni-wuerzburg.de (I.B.d.M.); florian.kai.groeber-becker@isc.fraunhofer.de (F.G.-B.); daniel.kruse@uni-wuerzburg.de (D.K.); 3 EVOMEDIS GmbH, 8036 Graz, Austria; martin.funk@evomedis.com; 4 Translational Center Regenerative Therapies, Fraunhofer Institute for Silicate Research ISC, 97080 Würzburg, Germany; 5 TPL Path Labs GmbH, 79111 Freiburg, Germany; kukla@tpl-path-labs.com (F.K.); lemarchand@tpl-path-labs.com (T.L.); 6 Joanneum Research Forschungsgesellschaft mbH, COREMED, 8036 Graz, Austria

**Keywords:** bacterial cellulose dressing, secondary wound dressing, moisture balance, wound healing, in vivo experiments

## Abstract

A balanced and moist wound environment and surface increases the effect of various growth factors, cytokines, and chemokines, stimulating cell growth and wound healing. Considering this fact, we tested in vitro and in vivo water evaporation rates from the cellulose dressing epicite^hydro^ when combined with different secondary dressings as well as the resulting wound healing efficacy in a porcine donor site model. The aim of this study was to evaluate how the different rates of water evaporation affected wound healing efficacy. To this end, epicite^hydro^ primary dressing, in combination with different secondary dressing materials (cotton gauze, JELONET^◊^, AQUACEL^®^ Extra ™, and OPSITE^◊^ Flexifix), was placed on 3 × 3 cm-sized dermatome wounds with a depth of 1.2 mm on the flanks of domestic pigs. The healing process was analyzed histologically and quantified by morphometry. High water evaporation rates by using the correct secondary dressing, such as cotton gauze, favored a better re-epithelialization in comparison with the low water evaporation resulting from an occlusive secondary dressing, which favored the formation of a new and intact dermal tissue that nearly fully replaced all the dermis that was removed during wounding. This newly available evidence may be of great benefit to clinical wound management.

## 1. Introduction

Controlling the homeostatic moist wound state is an essential element of wound care [1]. Considering the sequential phases involved in tissue repair, i.e., the inflammatory, proliferative, and regeneration phases, they occur at specific intervals after trauma. Generally, the proliferative phase begins within the first 48 h and lasts up to 14 days. It is responsible for closure of the wound and includes fibroplasia, angiogenesis, and re-epithelialization [2]. Thus, the healing process can be aided by maintaining moisture balance, which facilitates cellular growth and migration as well as the action of growth factors, cytokines, and chemokines [2,3,4]. As a result, faster and more effective re-epithelialization and revascularization are promoted [5]. A general increase in cellular proliferation can be seen in a moist wound environment, resulting in increased quantity and consistency of the extracellular matrix [6,7].

Furthermore, a liquid wound environment may improve cosmetic outcomes [8,9,10]. However, there is still a lack of clarification about the amount of hydration required to facilitate optimal healing and how different moisture environments influence tissue regeneration [11]. When considering a hyperhydration treatment, for instance, a common clinical side effect is a whitened, wrinkled, and moist appearance termed maceration, especially in the periwound area when it is not protected [12,13]. There have been several examples in the literature showing that damage caused by excessive hydration can be reversed [14,15,16].

Concomitantly, a modern wound dressing must meet several other criteria, including facilitating rapid and painless wound healing, maintaining a stable pH and temperature, and acting as a bacterial barrier. It should also be able to absorb exudates without drying the wound by keeping the environment moist [17,18]. Hydroactive bacterial nanocellulose (BNC) wound dressings are commercially available and meet these specifications. 

Determining a method of controlling the evaporation rate of a wound dressing could be the key to achieving different moisture balances and perhaps positively influencing the healing properties of the wounded tissue. As is known, evaporation characteristics vary depending on the wound dressing [19]. The aim of the present study was to test the effects of different secondary wound dressings used in clinical routine in combination with epicite^hydro^, in vitro and in vivo, in order to analyze the influence of moisture on the healing process of epidermal and dermal tissues.

## 2. Materials and Methods

### 2.1. Wound Dressings

For our experiments, we used a BNC-based hydroactive wound dressing (epicite^hydro®^, QRSkin GmbH, Würzburg, Germany) as the primary wound dressing. epicite^hydro^ is a biomaterial made of biotechnologically generated nanofibrillar cellulose synthesized by the bacterium *Komagataeibacter xylinus* DSM 14666 (culture collection of the Friedrich Schiller University Jena, deposited at the DSMZ, German Collection of Microorganisms and Cell Cultures, Braunschweig, Germany). It has an isotonic saline solution content of more than 95%; therefore, it is perfectly suited to provide the necessary moisture as a primary dressing. The secondary wound dressings used in this study were cotton gauze (Gazin^®^ Lohmann & Rauscher, Rengsdorf, Germany), a hydrocolloid dressing composed of sodium carboxy-methylcellulose fibers (AQUACEL^®^ Extra™, ConvaTec GmbH, Munich, Germany), a paraffin gauze (JELONET^◊^, Smith & Nephew GmbH, Hamburg, Germany), and a synthetic moisture vapor-permeable polyurethane film (OPSITE^◊^ Flexifix, Smith & Nephew GmbH, Hamburg, Germany). We exclusively chose wound dressings as secondary layers, which are often used in clinical routines.

### 2.2. In Vitro Dehydration Experiments

Before testing and evaluating in vivo experiments, we performed in vitro experiments to determine the different evaporation rates of the wound dressing that will be used in combination with the BNC. For this purpose, circular epicite^hydro^ samples in triplicate with diameters of 3 cm were weighed using a precision balance (Kern PCB 1000-2). The samples were left uncovered (control) or covered with different secondary dressings (each in triplicate) and placed at T = 32 °C in an incubator. The samples were weighed at 10 different timepoints, and the relative weight loss compared to the input material was calculated.

### 2.3. Porcine In Vivo Experiments 

The Animal Care and Use Committee (Veterinary University Vienna, Austrian Ministry of Science and Research) approved the animal experiments (protocol code 66.010/0039-WF/V/3b/2017, 04/01/2017). The animals were treated, under anesthesia and analgesia, with epicite^hydro^ in combination with one of the four different secondary dressings (cotton gauze, AQUACEL^®^ Extra™, JELONET^◊^ and OPSITE^◊^ Flexifix), which were placed on 3 cm × 3 cm-sized dermatome wounds with a depth of 1.2 mm arranged on the flank of a domestic pig (*Sus domesticus*) with three sites/applications each. A total of 12 wounds were generated per animal, 6 on each flank. After five days, the first animal was sacrificed, and photo documentation and tissue/wound dressing sampling for further analysis was performed. Another animal was used to confirm the results. The second animal was sacrificed after seven days in order to assess the effects of dressing modalities in slightly more mature wounds and confirm the preliminary results observed after five days. For the second animal, cotton gauze and OPSITE^◊^ Flexifix were used and contrasted with 4 sites/applications per treatment condition.

### 2.4. Microscopic Analysis of Wounds and Re-Epithelialization Assessment

For microscopic analysis, 8 mm punch biopsies from the wounds were carefully taken through the dressing and kept it in place. Afterwards, the remaining wound dressing was removed for analysis of the residual water content, and all wounds with a size of 3 × 3 cm and surrounded by 1 cm of healthy tissue were excised above the fascia. Tissue samples were fixed for 48–72 h with 4% formaldehyde, embedded in paraffin, and sectioned at a nominal thickness of 3 μm. Sections were stained with routine hematoxylin and eosin (H&E).

The study pathologist performed a blind histopathological analysis. The qualitative evaluation included scoring with a 4-grade scale (minimal, slight, moderate, marked), re-epithelialization, fibroplasia/neovascularization (granulation tissue amount and thickness), inflammation (relative amounts of polymorphonuclear leukocytes and pus, mononuclear cells and foreign-body multinucleate giant cells, amount of exudation), and degeneration/necrosis if appropriate. In addition, an estimation of the global percentage of re-epithelialization was performed by estimating a percentage of 16 to 21% immediately following 100× microscopic fields covering the entire wound section, with three wounds of each group.

Additionally, a scoring system was used to assess the density of inflammatory cells that infiltrated the new granulation tissue in the experiment after seven days of healing. Therefore, a 0 to 5 score grading was established based on different criteria (Table 1).

All densities were evaluated at approximatively the mid-depth of the new regenerating dermal (granulation) tissue; thus, it was anticipated that the densities of new global dermal tissues may differ slightly.

Lastly, all H&E slides were scanned using a calibrated Axioscan Z1 whole slide scanner (Zeiss, Jena, Germany), and a quantitative morphometry of re-epithelialization and new dermis was performed. Manual measurements included the wound width at the section level, the sum of the lengths of the epithelial gaps or the sum of the lengths of the newly formed epithelium, and percentage of re-epithelialization as the ratio of the total length of newly formed epithelium to the wound length. In addition, the average thickness of newly formed dermal tissue was determined using the Halo^®^ tissue classifier™ (Indica Labs, Albuquerque, NM, USA) on day 5 and the AI CNN-based tissue classifier (Visiopharm^®^, Hoersholm, Denmark) on day 7. Measurements on both days were confirmed manually by dividing the area of the new connective tissue by the wound length. All illustrations were prepared from whole digital sections.

### 2.5. In Vivo Dehydration Experiments

The epicite^hydro^ samples taken from the wounds at the end of the in vivo experiments were stored in dry ice cooling until testing of the residual water content. Wet sample weight (mw) (*n* = 3) was determined directly after defrosting using an analytical balance (Kern ABJ 220-4NM). Afterwards, samples were air dried at room temperature until the mass achieved constancy. Dry mass (md) (*n* = 3) of samples was determined by weighing (Kern PCB 1000-2). The remaining water content (WC) of epicite^hydro^ samples was calculated using the following formula: WC = ((mw − md)/mw) * 100%. To confirm these results, primary dressing average thickness (in µm) was measured through H&E-stained slides in triplicate (each slide was measured 15 times, for a total of 45 measurements per treatment). BNC dressings were not removed before fixation and posterior staining, and manual measurements of the primary dressing thickness were obtained using the Zen 3.3 Blue Edition (Zeiss Microscopy, Jena, Germany) image processing software.

### 2.6. Immunofluorescence Staining

Indirect immunofluorescence staining of the tissue sections was performed using a primary antibody specific to the High Mobility Group protein B1 (HMGB1, 1:100; Cell Signaling Technology, Beverly, MA, USA), followed by an incubation with a secondary antibody solution coupled with Alexa Fluor^®^ 555 (1:400; Life Technologies, Darmstadt, Germany) and a fluorescein isothiocyanate (FITC)-conjugated anti-α-smooth muscle actin antibody (ASMA, 1:100; Abcam, Cambridge, MA, USA). Cell nuclei were stained using 4′,6-diamidino-2-phenylindole (DAPI) in a Fluoromount-G DAPI mounting medium (Life Technologies, Darmstadt, Germany). Images were taken using a KEYENCE BZ 9000 microscope (Keyence, Neu-Isenburg, Germany) with 10× or 20× magnification.

### 2.7. Assessment of Blood Vessel Density through Imaging Software

Immunofluorescence staining of the tissue sections was performed using ASMA (1:100; ab8211, Abcam, Cambridge, MA, USA, and images were captured using an Axioscan Z1 whole slide scanner (Zeiss, Jena, Germany). Pictures were evaluated using Zen 3.3 Blue Edition (Zeiss Microscopy, Jena, Germany). The fluorescence channel for the α-smooth muscle actin (αSMA) was dimmed to enhance the blood vessel signal, and the density of the αSMA per mm² was measured using the ImageJ software, version 1.53e (developed by Wayne Rasband, National Institutes of Health, Bethesda, MD, USA), and Java 1.6.0_24 (64 bits). Counts matching 0.3 to 1.0 circularity were considered during our analysis.

### 2.8. Statistical Analysis

Data were analyzed in GraphPad Prism 8.0 (GraphPad Software, Inc., San Diego, CA, USA) through paired two-tailed T tests and Pearson correlations.

## 3. Results

### 3.1. In Vitro Dehydration Experiments

**Figure 1:**
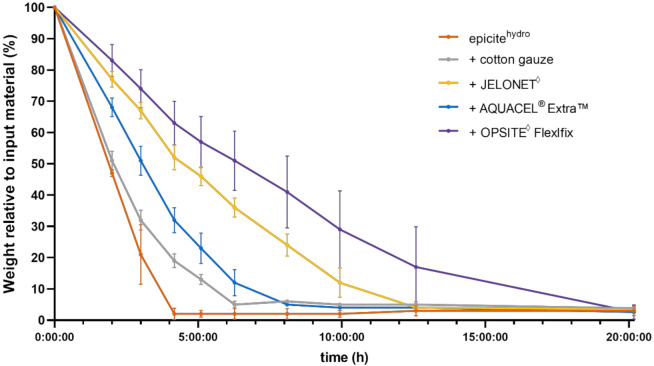
In vitro evaporation from epicite^hydro^ in combination with different secondary dressings. Weight of epicite^hydro^, relative to initial weight, after incubation at 32 °C for each timepoint. The BNC fleece was incubated without any coverage (orange) and with epicite^hydro^ + cotton gauze (grey), epicite^hydro^ + JELONET^◊^ (yellow), epicite^hydro^ + AQUACEL^®^ Extra ™ (blue), and epicite^hydro^ + OPSITE^◊^ Flexifix (purple).

Water evaporation data from epicite^hydro^ samples left uncovered or covered with different secondary dressings were analyzed during and after drying at 32 °C. The resulting weight loss profiles are shown in Figure 1.

After twenty hours of drying at the end of the experiment, all samples showed an almost complete water loss, as indicated by a remaining weight of 2 to 5%, corresponding to the cellulose material present in epicite^hydro^. All profiles displayed a similar trend evidenced by fast and linear weight loss in the first 3–4 h followed by a phase of slower water evaporation. The epicite^hydro^ samples without a secondary dressing were completely dry after 4 h. The secondary dressings decreased the evaporation from the epicite^hydro^ samples in the following order: cotton gauze < AQUACEL^®^ Extra™ < JELONET◊ < OPSITE^◊^ Flexifix. Evaporation rates were calculated for each treatment. epicite^hydro^ without any secondary dressing showed the highest evaporation rate (8.41 mg/cm² * h; SD: ±1.26), followed by epicite^hydro^ + cotton gauze (7.58 mg/cm² * h; SD: ±0.42), epicite^hydro^ + AQUACEL^®^ Extra™ (6.11 mg/cm² * h; SD: ±0.28), epicite^hydro^ + JELONET◊^◊^ (3.89 mg/cm² * h; SD: ±0.56), and epicite^hydro^ + OPSITE^◊^ Flexifix (2.59 mg/cm² * h; SD: ±0.57).

Coverage with epicite^hydro^ + OPSITE^◊^ Flexifix and epicite^hydro^ + JELONET◊^◊^ led to 3- and 2.4-fold reductions, respectively, in the evaporation rate compared with the uncovered control. epicite^hydro^ + AQUACEL^®^ Extra™ showed a 2-fold reduction, while cotton gauze had minor effect on the evaporation.

### 3.2. Dehydration on Wounds In Vivo and New Tissue Formation after Five Days

**Figure 2:**
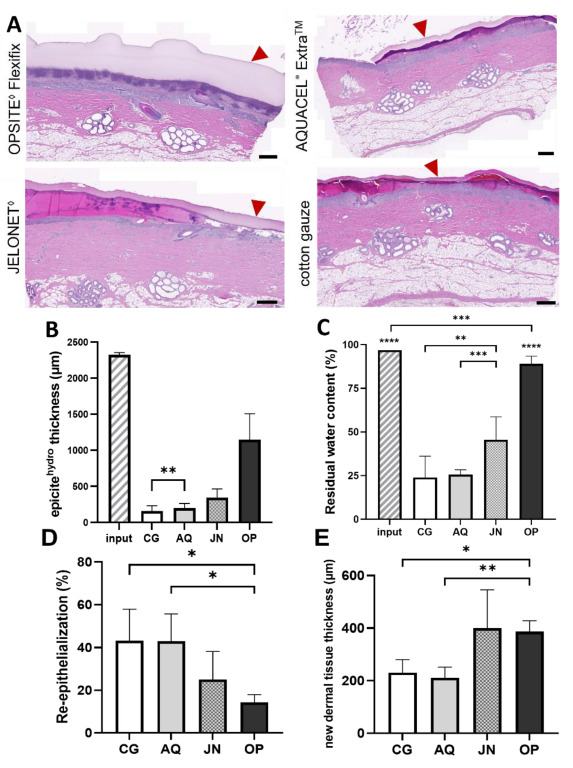
In vivo residual water analysis and healing process after five days of treatment. (**A**) H&E staining of punch biopsies extracted from the upper right area of the wound. Arrowheads highlight the remaining epicite^hydro^ present on the wounded area. The scale bar represents 500 µm. (**B**) Histogram of the thickness of the epicite^hydro^ combined with secondary wound dressing on the surface of the wound after five days of treatment. Statistical significance was obtained between all comparisons (*p* values: ** = 0.0037 and <0.001 for all other comparisons). (**C**) Histogram of relative residual water content of epicite^hydro^. All data were statistically different from input and from residual water of epicite^hydro^ combined with OPSITE^◊^ Flexifix. epicite^hydro^ + cotton gauze and epicite^hydro^ + AQUACEL^®^ Extra ™ showed differences in comparison with epicite^hydro^ + JELONET^◊^ (*p* values: ****: <0.0001; ***: 0.0009/0.0004; **: 0.0026). (**D**) Percentage of re-epithelialization observed at the wound area. Re-epithelialization was higher on the wounds treated with epicite^hydro^ + cotton gauze and epicite^hydro^ + AQUACEL^®^ Extra ™ than on those treated with epicite^hydro^ + OPSITE^◊^ Flexifix (*p* values: *: <0.05). (**E**) Average thickness of the new dermal tissue was obtained from the H&E slide measurement. Thicker tissue was formed on the wounds treated with epicite^hydro^ + OPSITE^◊^ Flexifix than on those treated with cotton gauze or AQUACEL^®^ Extra ™ as secondary dressing (*p* values: * 0.0298; **: 0.0064).

**Figure 3:**
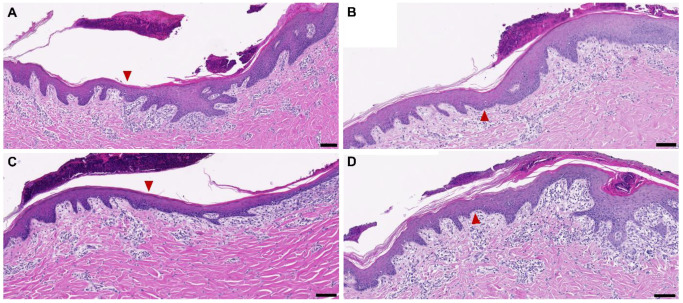
H&E staining of the wound edge area for the wounds after 5 days of treatment. Representative microscopies depicting the wound edge area after 5 days of treatment with epicite^hydro^ combined with cotton gauze (**A**), JELONET^◊^ (**B**), AQUACEL^®^ Extra ™ (**C**), and OPSITE^◊^ Flexifix (**D**). The red arrowhead roughly indicates the position of the wound edge. The scale bar represents 100 µm.

The microscopic evaluation of the primary dressing samples in combination with the different secondary dressings on donor site wounds of 1.2 mm depth after five days showed significant variability in remaining thickness and inferred liquid content. Samples covered with epicite^hydro^ + cotton gauze or epicite^hydro^ + AQUACEL^®^ Extra™ were the thinnest and hence were likely to be very dry, whereas epicite^hydro^ + JELONET^◊^ and epicite^hydro^ + OPSITE^◊^ Flexifix were slightly and considerably thicker, respectively, suggesting more residual liquid, as shown by H&E stainings (Figure 2A). Through a morphometric analysis of those stainings, the thickness of the remaining BNC dressing was measured (Figure 2B). A quantification of the residual water content (Figure 2C) was also performed and confirmed both previous results. The content of residual water matched the evaporation kinetics determined in vitro: epicite^hydro^ + cotton gauze (24%) < epicite^hydro^ + AQUACEL^®^ Extra™ (26%) < epicite^hydro^ + JELONET◊^◊^ (45%) < epicite^hydro^ + OPSITE^◊^ Flexifix (89%). Furthermore, the wound edges did not show any differences in the H&E stainings despite the different moisture content (Figure 3).

Pearson’s correlation analysis (Table 2) showed a very strong inverse relationship between evaporation rates found in vitro and residual water found in vivo after five days of treatment. When the relationship between the evaporation rates in vitro and the measured BNC thickness was calculated, a moderate to strong inverse relationship was obtained.

Next, a blinded microscopic evaluation of H&E-stained sections from wounds was performed to assess the healing effect for the different wound dressing combinations. After five days of treatment, the percentage re-epithelialization was significantly higher in the wounds treated with epicite^hydro^ + Aquacel^®^ Extra™ (43.0 ± 12.7%), and epicite^hydro^ + cotton gauze (43.2 ± 14.7%) than in those treated with an occlusive dressing (epicite^hydro^ + OPSITE^◊^ Flexifix—14.3 ± 3.6%) (Figure 2C). The opposite effect was observed regarding the formation of a new dermal tissue; significantly higher results were achieved by epicite^hydro^ + OPSITE^◊^ Flexifix (386.3 µm ± 41.6) and epicite^hydro^ + JELONET^◊^ (399.8 µm ± 146.1) than by nonocclusive (epicite^hydro^ + cotton gauze-231.1 µm ± 49.8 and epicite^hydro^ + Aquacel^®^ Extra™ 211.5 µm ± 40.4) dressings (Figure 2E).

### 3.3. Influence of Nonocclusive and Occlusive Dressing after Seven Days of Treatment

To focus the analysis on the comparison of the effects of nonocclusive and occlusive dressings after prolonged treatment, the wounds were treated for seven days using either cotton gauze or OPSITE^◊^ Flexifix as secondary dressings.

Comparing the results (Table 1) for the evaporation rate obtained in vitro and the water content remaining in the BNC dressing after seven days of treatment, a very strong and negative relationship was obtained.

**Figure 4:**
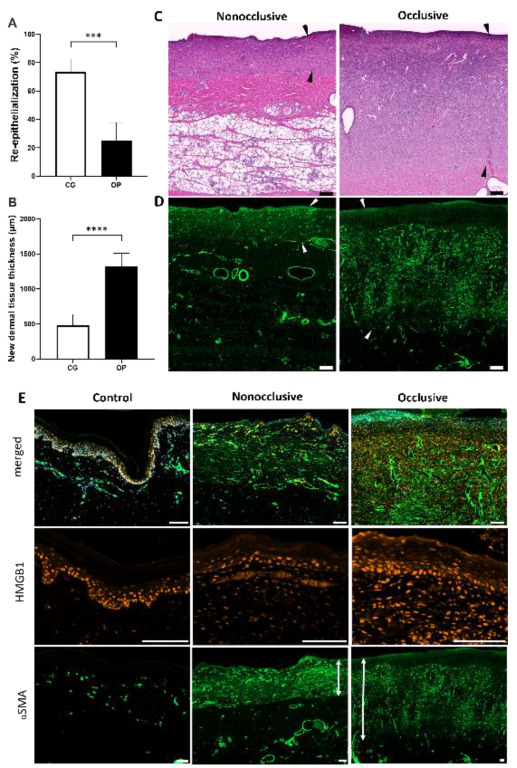
Wound healing process of the tissue after seven days of treatment. Percentage re-epithelialization for nonocclusive and occlusive dressings (**A**) and average thickness of new dermal tissue (**B**) obtained by the analysis of H&E staining. H&E staining (**C**) and immunofluorescence staining for anti-α-smooth muscle actin (αSMA, green) (**D**) of wounds treated with occlusive and nonocclusive secondary wound dressing. Red and white arrowheads indicate the epidermis on top and the end of regenerated dermis at the bottom. The scale bar represents 200 µm. Immunofluorescence staining of skin untreated and treated with nonocclusive and occlusive secondary dressings. Merged images labeled with DAPI (blue), high mobility group protein B1 (HMGB1, red), and αSMA (green) showed a staining pattern for the untreated skin (control) and the wounded skin treated with nonocclusive and occlusive dressing. HMGB1 staining showed undamaged cells under both treatments in comparison with the control group. αSMA staining revealed the presence of vessels in the tissue. The vertical doubled arrow lines indicate the thickness of regenerated dermal tissue containing activated myofibroblasts for both treatments (**E**). *p* values: ***: 0.0008; ****: < 0.0001. Scale bar: 100 µm.

The percentage of re-epithelialization achieved was significantly higher in the wounds treated with epicite^hydro^ + cotton gauze (73.5 ± 9.0%) than in those treated with epicite^hydro^ + the occlusive dressing OPSITE^◊^ Flexifix (25.1 ± 12.6%) (Figure 4A). However, the opposite effect was observed regarding the formation of a new dermal tissue, with significantly higher results being achieved by epicite^hydro^ + OPSITE^◊^ Flexifix (1316 ± 195 µm) than by nonocclusive dressing (epicite^hydro^ + cotton gauze—479 ± 159 µm) (Figure 4B).

A very strong positive relationship was found when the evaporation rate was compared with the percentage of re-epithelialization, while a very strong negative relationship was obtained for its correlation with the thickness of the newly formed dermis. 

The H&E staining of samples after the two treatments revealed a noticeable difference in the newly-formed dermal tissue (Figure 4C). The new tissue on the donor site treated with occlusive secondary dressing was massive and close to three times thicker than that on the donor site treated with nonocclusive dressing. In most cases, this filled up the entire portion of the removed dermal tissue, suggesting potential for a full recovery with the occlusive secondary dressing OPSITE^◊^ Flexifix.

Immunofluorescence staining for αSMA indicated the presence of activated myofibroblasts and a high number of blood vessels on the whole extension of the new dermal tissue, apart from a small area of macerated tissue on the surface of the wound treated with occlusive dressing (Figure 4D).

An anti-HMGB1 antibody was used as a marker for cells undergoing necrosis (Figure 4E). The presence of cells positive for HMGB1 nuclear staining in all analyzed samples indicated that the cells were not undergoing a necrotic process. Positive HMGB1 cells were found even in the tissue directly underneath the macerated tissue, where neither vessels nor activated myofibroblasts were observed.

**Figure 5:**
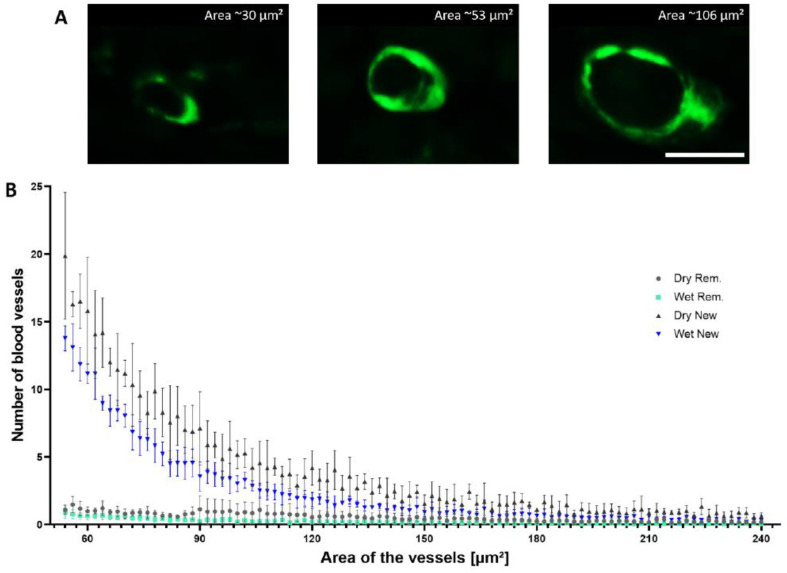
Assessment of blood vessel density through immunofluorescence image analysis. (**A**) Anti-αSMA immunofluorescence staining of blood vessels in the dermis of the analyzed tissue measured using imaging software, which revealed different sizes. (**B**) Density and distribution of blood vessels (per mm²) for the reminiscent tissue and the newly formed tissue treated with the occlusive (including superficial macerated tissue) and nonocclusive secondary dressing. The scale bar represents 10 µm.

To compare the blood vessel density in the tissues formed after different treatments, we decided to quantify the number and size distribution of blood vessels present in the reminiscent and new dermal tissues using an image processing approach (Figure 5) [20,21]. Hence, blood vessels positive for αSMA staining were counted using ImageJ, and the results were then plotted in a graph based on their sizes (in µm²) and density (per mm²). Both newly formed dermal tissue areas presented similar densities of small blood vessels (area < 250 µm²), which were found to be higher than that in the unwounded control area.

**Figure 6:**
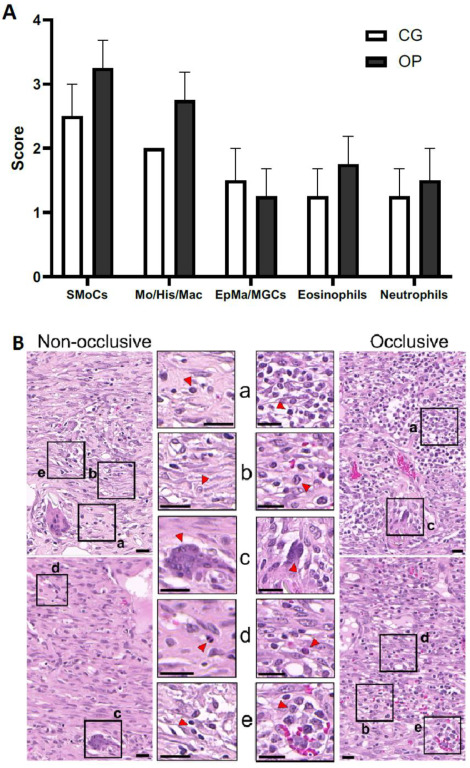
Histological aspects of new dermal tissue infiltrate composition. (**A**) Diagram with the pathology score, ranging from 0 to 5, for the presence of inflammatory cells in the newly formed dermal tissue: small- to mid-size mononuclear cell (SMC) (including lymphoblast) density; monocyte/histocyte/macrophage (Mo/His/Mac) density; epithelioid macrophage/multinucleate giant cell (EpMa/MGC) density; and granulocyte density, divided into eosinophils and neutrophils in both wounds treated with nonocclusive secondary wound dressing (cotton gauze—CG) and with occlusive dressing (OPSITE^◊^ Flexifix—OP). (**B**) Example of hematoxylin and eosin (H&E) sections with four representative sites of the two secondary dressings (nonocclusive and occlusive). Highlighted in a higher magnification (red arrowheads) are examples on both tissues of SMCs (**a**), Mo/His/Mac (**b**), EpMa/MGCs (**c**), and granulocytes (**d**) as well as the presence of mononuclear cells forming clusters (**e**). The scale bar represents 20 µm.

A qualitative scoring of inflammatory cell density in the mid layers of the new dermal tissue (Figure 6) also revealed similar results. Small mononuclear cells (including mainly small lymphocytes but also plasma cells) had a higher density in the occlusive-covered than in the nonocclusive-covered wounds. Qualitative scoring revealed similar epithelioid and multinucleate giant cell responses around cotton fibers and/or other debris in both types of wound dressing. Additionally, these mononuclear cells of small and medium size (including cells with morphology that suggested the presence of lymphoblasts) were more often observed in small or large clusters in the occlusive-covered wound sites. However, it was not possible to qualitatively know if the mitosis density increased or if this was simply related to a new and more abundant granulation tissue.

## 4. Discussion

### 4.1. Comparison of In Vitro and In Vivo Water Evaporation Results 

In our experiments, we were able to show, both in vitro and in vivo, the differences in the evaporation characteristics of the cellulose wound dressing epicite^hydro^ when combined with different secondary dressings available on the market. Recently, Xu et al., using a polyurethane membrane with a water vapor transmission rate (WVTR) of 2028.3 ± 237.8 g/m^2^·24 h, obtained a moisture content sufficient to support the sizable proliferation and regular functioning of epidermal cells and fibroblasts in a mouse skin wound model [22]. However, the results were obtained after controlling only the evaporation rate of the moisture present in the wound bed without providing additional water. The BNC used in the present work offered a source for wound hydration. Furthermore, in wounds with strong exudation, BNC has the capacity to absorb it. This has been already documented in the current literature [23]. The evaporation capacity of the BNC can be easily controlled through combination with a secondary dressing. To put this into perspective, our in vitro tests showed WVTRs of 2019.3 ± 302.0 g/m^2^·24 h for epicite^hydro^ without coverage, 1819.2 ± 101.9 g/m^2^·24 h for epicite^hydro^ + cotton gauze, 1466.8 ± 67.9 g/m^2^·24 h for epicite^hydro^ + AQUACEL^®^ Extra^TM^, 933.7 ± 134.8 g/m^2^·24 h for epicite^hydro^ + JELONET^◊^, and 621.3 ± 135.8 g/m^2^·24 h for epicite^hydro^ + OPSITE^◊^ Flexifix. Lachenbruch et al. showed similar evaporation rates for some of the wound dressings tested. They showed a high retention of water when using OPSITE^◊^ Flexifix and a fast evaporation rate when using cotton gauze; these findings were in full agreement with our results [19].

Residual moisture observed in the dressings after removal from wound sites in the swine model at the end of the treatment in our in vivo tests (five days and seven days) were highly consistent with those previously found in in vitro tests.

### 4.2. Moisture Balance on Occlusive Secondary Wound Dressing

Our results show differences in wound healing by controlling the evaporation rate from the wound by combining epicite^hydro^ with different secondary dressings. Wound hydration is now the basis of modern wound care and plays a pivotal role in preventing the desiccation of wound surfaces and deeper wound layers [11,24,25,26]. Excessive hydration is often not recommended; however, experiments showed that when exposing skin tissue to water for 72 and 144 h, only mild dermatitis was observed [27]. Other studies reported that prolonged water exposure had effects that were not necessarily considered as damaging or adverse, such as increased epidermal thickness and the dilatation of intracellular spaces coupled with the swelling of the stratum corneum [28,29]. Thus, the hyperhydration of the skin with isotonic fluids should not always be necessarily considered as harmful [28]. In our experiments using epicite^hydro^ with OPSITE^◊^ Flexifix, the observed effect in the treated tissue was due to prolonged exposure with a high grade of occlusion of the wounds to the wound exudate and the isotonic fluid contained in the BNC dressing. The risk of infection could also be elevated in an extended application. However, the combination of BNC dressings with antiseptics for a sustained delivery has been well described [30,31] and could be applied if necessary. While conducting our experiments, no infections were observed after five and seven days of treatment.

### 4.3. Wound Healing Effect after Different Treatment Times 

Wound healing experiments were performed in a porcine wound model. Animal wound models are widely accepted for preclinical experiments in this scientific field [32]. Because of the similarities between human and porcine skin, this wound model provides an accurate option to investigate wound healing [33]. Furthermore, the use of models can represent the possibility and feasibility of simulating clinical scenarios [34]. Using porcine models, Winters and Scales demonstrated the positive impact of a moist environment during wound healing [35]. Hinman and Maibach followed by reaching similar results in split thickness wounds from volunteer human donors [36].

Our findings showed that the application of an occlusive secondary wound dressing promoted the formation of a granulation tissue over the re-epithelialization process, whereas the combination with a secondary nonocclusive dressing favored opposite processes: lower granulation tissue and earlier re-epithelialization. Many experiments, however, have shown that dry wound healing can slow down re-epithelialization by forming a scab [24,28]. Moisture balance is recognized in the wound care field as crucial for achieving better wound healing [22,35,36], and our results demonstrated that moisture conditions were most favorable for reconstructing abundant new dermis. An early re-epithelialization may be less favorable in enabling full dermal reconstruction by new granulation tissues [37].

Treatment time seems to amplify the differences between these processes. When the wounds were treated for seven days, in comparison with treatment for five days, the re-epithelialization process was around 1.7 times more efficient. This difference was more pronounced for nonocclusive secondary dressing (Δ_(seven days−five days)_ = 30.3%) than for occlusive secondary dressing (Δ_(seven days−five days)_ = 10.9%). Similarly, the thickness of the new dermal tissue was increased when treatment was prolonged. A massive difference was observed, around 3.4 times higher, when an occlusive secondary dressing was used (Δ_(seven days−five days)_ = 929 µm) and a thickness around 2.0 times higher was observed for nonocclusive dressings (Δ_(seven days−five days)_ = 247 µm). There was even an improvement in the correlation coefficient for the evaporation rates obtained in vitro and the thickness of the newly regenerated dermal tissue. After five days of treatment, the relationship between these two parameters was moderate, whereas after seven days, the relationship established was very strong. These results pointed to a positive effect for re-epithelialization of prolonged treatment with BNC dressing in combination with a nonocclusive dressing. Similarly, new dermal tissue formation seems to benefit when longer treatment with BNC in combination with occlusive dressing is established.

### 4.4. Characteristics of the Newly Formed Dermal Tissue

In both occlusive and nonocclusive dressings, the new dermal tissue was fully within normal limits and had no notable qualitative differences except that the small- to medium-sized mononuclear cell density at mid-depth of the new dermal tissue was minimally to slightly higher in wounds covered with occlusive dressing (epicite^hydro^ + OPSITE^◊^ Flexifix) than in those covered with nonocclusive dressing (epicite^hydro^ + cotton gauze).

Activated myofibroblasts expressing αSMA are considered to be a hallmark for maturing new dermal tissue [38]. These cells were observed, correlating with the H&E staining, throughout the maturing new dermal tissue, down to the deeper layers of the wounds treated with nonocclusive or occlusive secondary dressing in combination with the BNC dressing. Activated myofibroblasts were absent only on the surface of the wounds treated with occlusive secondary dressing, underneath the layer with relatively abundant exudate, which is to be expected in active proliferating regenerating connective tissue.

Marker proteins, such as HMGB1, can offer a perspective on the stress level that cells face in the new tissue and indicate if they are undergoing a degenerative/necrotic process [39,40]. Apart from the thin upper layer of tissue (i.e., the transition layer above the proliferative layer) located directly under the relatively abundant exudate of the wounds treated with occlusive secondary dressing, both treatments and the control showed positive results for this marker, indicating that the cells were not under stress after seven days. This result is especially important since it suggests that no additional stress was observed under occlusive conditions. The high density of HMGB1-positive cells in both treated and new dermal tissues was due to the infiltration of the inflammatory cells; the protein stained by this marker was ubiquitously expressed [39]. In the zone directly under the minimally macerated tissue, cells positive for the marker were still found in layers with a lack of nearby vessels and activated myofibroblasts, as evidenced by the αSMA staining.

Blood vessel quantification showed similar density in both treatments after seven days, notwithstanding the fact that the superficially macerated tissue and/or exudate containing degenerated inflammatory cells were included in our analysis. Therefore, we considered that, despite the difference in the average thickness of new granulation tissue for both treatments, combinations with occlusive and nonocclusive secondary dressing offered similar vascularization. This finding may be important for clinicians in their consideration of approaches to different wound healing scenarios.

Overall, small inflammatory/immune cells, mainly lymphocytes and possibly monocytes (immature macrophages), were of minimally to slightly higher densities; thus, they were more numerous in thicker, occlusive-treated wound sites, while other cell types were not substantially different. Mononuclear cells such as lymphocytes and other innate-immunity cells are known to favor more active and new dermal tissues because of the numerous cytokines they secrete [3,41].

## 5. Conclusions, Limitations and Clinical Aspects

In the present study, we were able to show that a high-moisture content dressing could influence different healing processes depending on its combination with a secondary wound dressing. Combination with a nonocclusive dressing could support the re-epithelialization process, whereas combination with an occlusive secondary wound dressing significantly promoted the regeneration of granulation/new dermal tissue. In our experiments, the granulated tissues showed no disadvantageous characteristics concerning tissue quality (e.g., blood vessel density) or in other cell types.

Of course, one limitation could be that this study represents wounds with a size of 3 cm × 3 cm, which can seem small when considering healing of larger defects. Nevertheless, this study can indicate which dressing could be used for larger defects. However, further studies and data are needed to clarify this question. Another limitation arises when the question of scarring is raised. However, in order to assess tissue scarring, wounds must be observed over a longer period of time, and a different staining protocol must be used. Therefore, with the results obtained so far, it is not yet possible to predict the outcome of scarring.

From a clinical point of view, these results should be considered as an important and useful tool for the treatment of patients with burns or chronic wounds. Based on the effects observed in this study regarding the regeneration of the dermis (granulation tissue), we can see how important it is to choose the right wound dressing, or combination of different modern wound dressings, to achieve the various necessary effects in different wound stages. A deeper wound would be likelier to benefit from a more occlusive dressing until sufficient dermal restoration has occurred, followed by a nonocclusive dressing to facilitate re-epithelialization in the later stages. This means that, depending on the type of wound and the individual needs of the patient, a buildup of the dermis can take place before any re-epithelialization. In this way, the body’s natural regenerative mechanisms can be optimally utilized or appropriately supported by correct choices in dressing materials.

## Figures and Tables

**Table 1:** Scoring for density of inflammatory cells.

	Score Description
0	None in tissue
1	Very low density in the new dermal tissue at around mid-depth; a few cells could be found when roaming through the tissue
2	Low density of these cells in the new tissue; 1 to 5 cells may have been found at high magnifications of 200× or 100% of the scan at mid-depth
3	Significant intermediate density, or around many vessels; 6 to 20 cells noted in about every high-power field (200× or 100% magnification)
4	High density; at least 21–100 cells in about every high-power field; may focally partially obliterate (erase) the new dermal tissue
5	Coalescing to diffuse (e.g., a lymph node for small mononuclear cells or a granuloma for epithelioid macrophages/multinucleate giant cells), almost completely erasing the new “granulation tissue”

**Table 2:** Pearson’s correlation results for the relationship between in vitro and in vivo efficacy parameters. Results are presented as Pearson’s coefficient r at a 95% confidence interval.

	Five Day Treatment				Seven Day Treatment		
	Residual Water In Vivo	BNC Thickness In Vivo	% Re-Epithelial	New Granulation Tissue	Residual Water In Vivo	% Re-Epithelial	New Granulation Tissue
Evaporation rates in vitro	−0.87 *	−0.79 *	0.76 *	−0.71 *	−0.84 *	0.93 *	−0.93 *
	−0.96 to −0.0	−0.94 to −0.40	0.32 to 0.93	−0.91 to −0.22	−0.97 to −0.34	0.66 to 0.99	−0.99 to −0.64
Residual water in vivo		0.90*	−0.63 *	0.70 *		−0.86 *	0.85 *
		0.70 to 0.97	−0.89 to −0.09	0.20 to 0.91		−0.97 to −0.39	0.36 to 0.97
BNC thickness in vivo			−0.70 *	0.54			
			−0.91 to −0.20	−0.048 to 0.85			

* Statistically significant (*p* < 0.05) for Pearson’s analysis.

## Data Availability

The data presented in this study are available on request from the corresponding author.
